# Clinical, Economical, and Organizational Impact of Chronic Ischemic Cardiovascular Disease in Italy: Evaluation of 2019 Nationwide Hospital Admissions Data

**DOI:** 10.3390/ijerph22040530

**Published:** 2025-03-31

**Authors:** Filomena Pietrantonio, Ciro Carrieri, Francesco Rosiello, Federico Spandonaro, Antonio Vinci, Daniela d’Angela

**Affiliations:** 1 Internal Medicine Unit, Medical Area Department, Castelli Hospital, Asl Roma 6, 00040 Roma, Italy; 2 Faculty of Medicine and Surgery, St. Camillus University of Health Sciences, 00131 Rome, Italy; 3 Centro per la Ricerca Economica Applicata in Sanità (C.R.E.A. Sanità), Università degli Studi di Roma Torvergata, 00133 Roma, Italy; c.carrieri@creasanita.it (C.C.); f.spandonaro@creasanita.it (F.S.); d.dangela@creasanita.it (D.d.); 4 Department of Infectious Disease and Public Health, Sapienza-University of Rome, 00185 Roma, Italy; 5 Local Health Authority ASL Roma 1, 00193 Roma, Italy; antonio.vinci@aslroma1.it

**Keywords:** internal medicine, cardiology, geriatrics, epidemiology, disease costs, hospitalization, chronic ischemic cardiovascular disease, CICD gender-based differences

## Abstract

Background: Chronic ischemic cardiovascular disease (CICD) is a common cardiovascular disease and a frequent cause of hospitalization, with significant differences between men and women. It is also an important comorbidity, affecting hospitalization length and mortality. The purpose of this paper is to investigate the clinical and economic impact of CICD on hospital admissions of non-surgical patients. Methods: To conduct the study, hospital discharge data (SDO) from each public and private hospital facility regularly sent by the regions to the Ministry of Health were analyzed, focusing on internal medicine, cardiology, and geriatrics departments’ 2019 discharged data coming from all Italian hospitals. Data were stratified according to age, gender, hospital charge ward, and costs. Results: The typical CICD patient is elderly (average age 80 years) and stays longer (+10.5 days) compared to the average one. They are also typically chronic patients with many comorbidities (respiratory and renal failure, as well as atrial fibrillation) in geriatrics and internal medicine departments, while in the cardiology departments, atrial fibrillation and outcomes of acute cardiovascular events predominate. Conclusions: CICD is a condition that leads to more hospitalizations in internal medicine departments than in cardiology and geriatrics departments and generates an average hospitalization value in line with the average one in internal medicine and geriatrics departments. In cardiology, the average value level is higher than the department average. Gender differences were found in cardiology departments; this data could suggest that the existing guidelines are affected by studies carried out mainly on males which lead to fewer recommendations for interventional procedures on females.

## 1. Introduction

Chronic ischemic cardiovascular disease (CICD) is one of the most common conditions worldwide, and a frequent cause of hospitalization in patients over 65, with significant differences observed between men and women. It is also a critical comorbidity, influencing both the length of hospital stay and patient mortality [1].

CICD originates from impaired coronary circulation, typically caused by the formation of atherosclerotic plaques in the coronary arteries [2]. A transient mismatch causing reversible myocardial ischemia is its dominant feature; CICD is also characterized by stable symptoms over a period of months, years, or even decades [3]. Stable angina is CICD’s most frequent presentation; other clinical presentations are microvascular angina, vasospastic angina, and ischemic cardiomyopathy [4,5]. Several risk factors are linked to CICD, including hypertension, diabetes mellitus, dyslipidemia, chronic obstructive pulmonary disease, smoking, and genetic predisposition. Adopting a healthy lifestyle, managing risk factors, and regular monitoring are all crucial for CICD prevention [6,7,8].

The staples of CICD treatment are myocardial perfusion improvement, symptom alleviation, and prevention of complications [9]. Therapeutic options include risk factors management, pharmacological therapy, revascularization procedures such as coronary angioplasty, surgical interventions such as coronary artery bypass grafting, and cardiac rehabilitation [1,10,11].

The prognosis of CICD depends on the severity of the disease and the effectiveness of the treatment. Proper management can enhance quality of life, reduce the risk of cardiac events, and prolong survival [12]. However, CICD requires long-term monitoring and continuous management to prevent disease progression [13,14].

The direct costs of hospitalization for CICD can vary significantly depending on various factors, including geographic region, hospital infrastructure, disease severity, length of stay, and specific treatments administered [15]. The patient population admitted to internal medicine departments, which largely consists of elderly individuals with multiple coexisting chronic conditions—often exacerbated and accompanied by critical episodes of clinical instability and functional organ deficits—means that real-life cases are often excluded from the guidelines designed for individual pathologies, as these do not reflect the evidence of large clinical trials [16].

Especially after the COVID-19 pandemic, whose toll was heavy on both territorial care and acute hospital facilities, the importance and role of internal medicine’s adequate management has been strengthened [17,18]. Since cardiovascular diseases represent a worldwide leading cause of death, and are preventable through lifestyle modifications, we decided to focus on their economic and organizational impact [19].

Since the main focus is on inpatient management, this study does not consider the impact on emergency services, including the activities carried out in the Emergency Department (ED) that do not result in acute care hospital admissions, nor does it cover outpatient, day-care, or home-based services [20].

The objective of this study is to describe the epidemiology of patients with CICD admitted to internal medicine departments that manage this condition (internal medicine, cardiology, geriatrics), assessing potential discrepancies in terms of comorbidities, length of hospitalization, and epidemiological characteristics [21,22]. In particular, comorbidities and age and gender differences were analyzed as they were previously linked with differences in treatment and outcome of cardiovascular diseases [23].

To conduct the study, hospital discharge data (SDO) from each public and private hospital facility regularly sent by the regions to the Ministry of Health were analyzed.

Furthermore, in Italy, internal medicine has dedicated departments, as do geriatrics and cardiology, and admission to each of the three departments occurs based on specific patient characteristics.

## 2. Materials and Methods

### 2.1. Study Design, Population, and Data Sources

This is a retrospective ecological study conducted using an existing national administrative database. The data used pertain to all hospital admissions in the designated departments (internal medicine, geriatrics, cardiology) of public and private healthcare facilities across Italy in the year 2019.

This investigation approach is quite standard in population-based studies, and has already been employed in several other nationwide studies [24].

The data were collected anonymously from the Hospital Discharge Records (Schede di Dimissione Ospedaliera, SDO) database managed by the Italian Ministry of Health.

The SDO database system was established by the Ministerial Decree of 28 December 1991 (GU Serie Generale n.13 del 17-01-1992 https://www.gazzettaufficiale.it/eli/id/1992/01/17/092A0166/sg (accessed on 9 February 2025), as a standard for gathering information on every discharged patient, from either public or private healthcare institutions, across the entire national territory. As Italian National Healthcare service is a Beveridge system, the SDO database is based on the ICD-9-CM classification system, coupled with the Diagnosis-Related Group (DRG) coding system.

The collected data include demographic characteristics of the patients (age, gender, residence, education level), hospitalization details (institution and specialty at discharge, admission type, discharge method), and clinical characteristics (primary diagnosis, comorbidities, diagnostic or therapeutic procedures).

The data were extracted by distinguishing between the primary diagnosis (the main cause for admission) and secondary diagnoses (conditions present that were not the direct cause of hospitalization but potentially contributed to clinical and/or organizational complexity).

SDO data were used to extract data related to cardiovascular diagnoses from internal medicine, cardiology, and geriatrics departments. Data from other departments were excluded, as the focus of this study is the evaluation of complex patients. According to the definition adopted by the Agency for Healthcare Research and Quality (AHRQ), a complex patient is a person suffering from two or more chronic diseases, in which each of the morbid conditions present is able to influence the outcome of the treatments of the other co-existing ones. This may happen through various modalities: limitation of life expectancy, increased intercurrent morbidity, interactions between pharmacological therapies, impossibility of full use of adequate treatments due to contraindications [13,25].

Since the main focus is on inpatient management, this study does not consider the impact on emergency services, including the activities carried out in the Emergency Department that do not result in acute care hospital admissions, nor does it cover outpatient, day-care, or home-based services.

### 2.2. Statistical Analysis

Data related to hospitalization episodes across the three ward types were presented in aggregated form, categorized by department type, the proportion of patients with CICD, and by gender. Mean values and percentages were reported as appropriate. The economic output generated by the DRG was calculated using the reimbursement rates provided by the National Health System for each hospital admission episode.

Results were processed and presented descriptively; since data from the entire population were available, no inferential analysis was required.

Excel v.16 MSO and STATA v.17 software were used for statistical analysis.

## 3. Results

### 3.1. Hospitalizations

**Figure 1:**
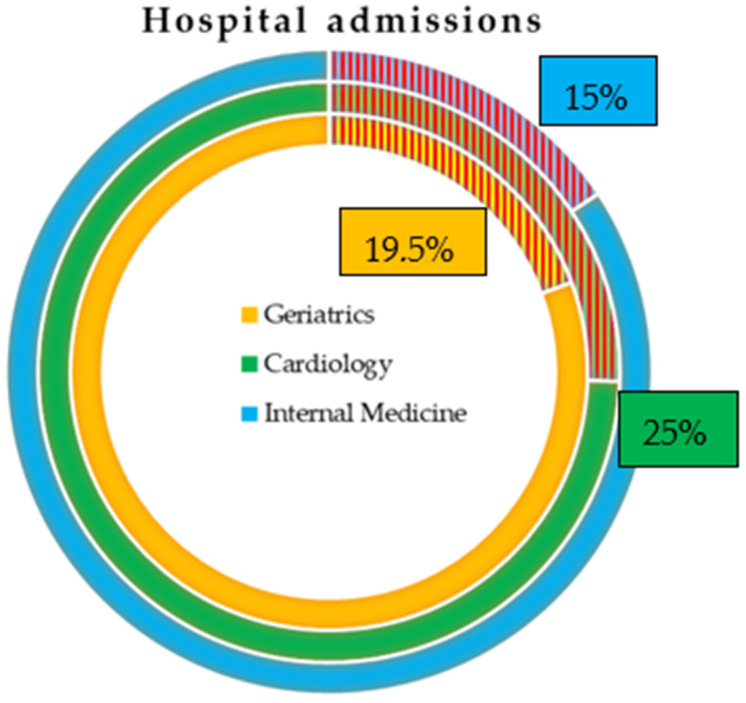
Proportion of CICD admissions in Italy in 2019. CICD represents 15% of internal medicine, 19.5% of geriatrics, and 25% of cardiology admissions. Source: processing of SDO data—© C.R.E.A. Sanità.

In 2019, the internal medicine, cardiology, and geriatrics wards generated 1,593,446 hospital admissions. Of these, 18.8% had a diagnosis of CICD (Figure 1).

Figure 1 represents the percentage of CICD admissions in Italy in 2019 according to the data provided by the National SDO Report. CICD represents 15% of internal medicine, 19.5% of geriatrics, and 25% of cardiology admissions. The three shaded areas represent the percentages of patients hospitalized with a diagnosis of CICD in each of the examined departments.

CICD accounted for 15% of hospitalizations in internal medicine, 19.5% in geriatrics, and 25% in cardiology. The highest number of patients was admitted to internal medicine (157,000), compared to 118,000 in cardiology and 25,000 in geriatrics. In cardiology, CICD was recorded as the primary diagnosis in almost half of cases; in internal medicine and geriatrics, this figure was close to one-third (Table 1).

While the highest proportion of CICD hospitalizations was observed in cardiology departments, followed by geriatrics, the largest volume of cases occurred in internal medicine. The average length of stay for CICD patients slightly exceeded the overall departmental averages: 10.5 days compared to 10.0 in internal medicine, and 6.8 days versus 5.9 in cardiology. The average length of stay was longer in the geriatrics and internal medicine departments, particularly in cases where CICD was not the primary diagnosis but rather a comorbidity.

Patients with CICD tended to be older than the average patient in these departments. In internal medicine, CICD patients had an average age of 81 years, compared to a departmental average of 75; in cardiology, the average age was 72 years compared to 69; and in geriatrics, CICD patients had an average age of 85 years, compared to a departmental average of 84 (Table 1).

Internal medicine and geriatrics wards had a similar pattern of comorbidities, with the most frequently observed condition being acute respiratory failure (12.8% of cases), followed by atrial fibrillation (10.3%), pneumonia and bronchopneumonia (6.6%), chronic acute respiratory failure (4.7%), exacerbated COPD (3.4%), and chronic renal failure (2.9%). In contrast, cardiology hospitalizations exhibited a different pattern of comorbidities, with the most common being atrial fibrillation (10.9%), left heart failure (6.2%), coronary atherosclerosis (6.1%), benign essential hypertension (6.0%), and diabetes (5.3%).

### 3.2. Economic Impact

The average cost per admission for patients with CICD is aligned with the overall ward averages in internal medicine (EUR 3527 vs. EUR 3505) and geriatrics (EUR 3640 vs. EUR 3650). In cardiology, however, the average cost per admission is higher, both in absolute terms and relative to the department’s average (EUR 5760 vs. EUR 5280) (Table 2 and Table 3).

In terms of total annual production generated by internal medicine, amounting to EUR 3,516,982,171, the portion attributed to CICD hospitalizations is 15% (EUR 555,264,058). Most of this expenditure was incurred for patients with CICD as a secondary diagnosis (EUR 411,301,495). Similarly, in geriatrics, with a total production of EUR 462,241,085, the share related to patients with CICD as the primary diagnosis was much smaller (5%) compared to those with CICD as a secondary diagnosis (14.3%). Overall, the expenditure for CICD amounted to EUR 89,282,223. In cardiology, however, with a total production of EUR 2,447,596,863, the distribution of costs between patients with CICD as the primary and secondary diagnosis was more balanced (12.3% vs. 15.5%), with a total expenditure of EUR 681,416,923 for CICD.

### 3.3. Organizational Impact on Healthcare Facilities

The progressive aging of the Italian population will likely lead to an increase in the volume of healthcare services related to both acute and chronic cardiovascular illnesses. However, in recent years, Italy has adopted health policies aimed at reducing behaviors which are known risk factors for ischemic heart disease, such as smoking, physical inactivity, alcohol consumption, and unbalanced diets. These factors still persist in the country, with significant North–South disparities and a notable impact on the younger population [26].

Compared to the pre-pandemic period, there has been a gradual reduction in hospital admissions due to acute myocardial infarction. Similarly, the number of coronary artery bypass graft surgeries has decreased, although mortality rates have remained stable. Conversely, evidence of a shift toward a population with a higher prevalence of chronic conditions is reflected in the increasing number of valvuloplasty or valve replacement procedures, except during the pandemic period (2020–2021), likely due to the reprioritization of resources in healthcare facilities. In Italy, despite significant improvements in the emergency care system, the reduction in the number of hospital beds over the last 30 years, and the increase in the number of fragile patients have led to an increase in ED attendance, a significant proportion of which are inappropriate. This means that many chronically ill patients are directed toward services that are structured to provide for acute care, but end up being clogged by patients whose health necessities are not otherwise provided, such as in Emergency Departments [27]. These inappropriate attendances drive up costs and increase system inefficiency [28,29].

The current high prevalence of chronic ischemic heart disease in the Italian population, driven not only by the absolute rise in incidence due to aging but also by improved diagnostic and therapeutic capabilities that allow patients to survive acute phases, places a significant burden on healthcare facilities. Beyond the increased hospitalizations in medical wards, the impact on surgical activities should not be overlooked, with the potential need to upgrade service delivery settings for patients with chronic ischemic heart disease. Additionally, there is the potential for discharge challenges, as patients may require transfer to intermediate care facilities equipped to manage complex cases, necessitating an integrated care approach [18].

### 3.4. Gender Inequalities

According to the AHA Scientific Statement 2016, from 1979 to 2011, ischemic heart disease mortality was higher in women than in men, in line with European data, where cardiovascular disease prevalence is greater in women (51%) compared to men (42%). This trend is also evident in Italy, although with slightly lower percentages. According to the literature [30,31], women who develop CICD have a higher risk of dying from the disease compared with their male counterparts. Mortality from CICD has decreased substantially among European Union countries. However, the declines were accompanied by a persistent gender gap in CICD mortality. One potential contributing factor to this gap is physical inactivity.

Acute myocardial infarction (AMI) is the leading cause of death in women worldwide, significantly worsening quality of life and leading to a high degree of morbidity due to heart failure. The clinical and instrumental approach to AMI and CICD are heavily influenced by studies conducted on male populations, with their findings indiscriminately applied to females. This approach fails to account for gender-specific differences that significantly influence the pathophysiology and clinical presentation of these conditions.

In Italy, we found that in cardiology wards, women over 65 with a diagnosis of CICD represent about half the number of men over 65 (27,893 vs. 58,049), a situation that does not occur in internal medicine and geriatrics. In internal medicine, there are 70,256 women over 65 compared to 75,120 men, while in geriatrics, there are 12,893 women and 11,420 men. For patients under 65, women are consistently fewer than men, representing half the number of men in internal medicine and geriatrics, and only about one-fifth in cardiology (Table 4). It is evident that due to gender issues, women tend to be admitted to the cardiology wards less frequently and are therefore subjected to interventional treatments less frequently than men.

It appears, therefore, that women undergo fewer revascularization procedures, which typically lead to hospitalization in cardiology, despite having similar comorbidity profiles to men. This disparity could be attributed to scientific evidence and, consequently, clinical guidelines and recommendations that are primarily based on studies involving predominantly male subjects.

Regardless of age or gender, heart failure is treated predominantly and at twice the frequency in internal medicine and geriatrics departments compared to cardiology.

In cardiology, the length of hospital stay for women over 65 is, on average, one day longer than for men, and 1.5 days longer in the under-65 age group, with an additional cost of approximately EUR 500. These gender-based differences in length of stay are not observed in geriatrics or internal medicine, where the average hospital stay for patients over 65 does not show significant gender disparities.

## 4. Discussion

### 4.1. Interpretation and Generalizability

The study carried out provides us with a national panorama characterized by the growing presence of elderly adults affected by CICD who are increasingly treated in the internal medicine wards as they are affected by comorbidities and not only by cardiac pathology, as requested by the cardiology specialists.

Management of chronic conditions is an ever-evolving challenge, especially in acute care settings and internal medicine wards. [32,33]. While many efforts are being directed in effective management of acute conditions, emphasizing both organizational and technological solutions, impact of chronic conditions on health systems as a whole is ever increasing, with difficulties in providing adequate care, especially in overburdened facilities [17,34].

On the other hand, the best of care to be provided is routinely established by evidence-based elements, and as such, the problem of gender bias in research may affect the day-to-day clinical practice [16]. In patients with cardiovascular diseases, timely identification and evaluation are fundamental for adequate treatment based on gender, severity, state of illness, and for risk reduction [20].

Nowadays, the typical profile of a medical patient with CICD is characterized by advanced age (average age 80) significant comorbidities, and an extended average length of stay (10.5 days). The average hospitalization value for CICD aligns closely with the average values in internal medicine and geriatrics; however, in cardiology, this average is significantly higher. CICD has a substantial impact on internal medicine, cardiology, and geriatrics, accounting for between 15% and 25% of both the number of hospitalizations and the economic value of activities. The total hospitalization costs amount to EUR 1.3 billion, with over 40% (EUR 555 million) occurring in internal medicine.

In cardiology, women over 65 with a diagnosis of CICD represent about half the number of men over 65, a pattern not observed in internal medicine and geriatrics. This disparity may suggest that existing guidelines are influenced by studies predominantly conducted on male samples, leading to fewer recommendations for interventional procedures in females.

### 4.2. Strengths and Limitations

This study uses data from a comprehensive national administrative database, providing a robust and representative dataset of hospital admissions across Italy for patients with CICD. This enhances the significance and generalizability of our findings, allowing us to derive insights into the clinical and economic impacts of CICD across the different medical specialty wards. Furthermore, the study design facilitates an exploration of gender disparities in hospitalizations and treatments, a critical area that remains under-researched in the context of cardiovascular disease. The retrospective nature of the study, coupled with rigorous data collection methods, contributes to the accuracy and reliability of the reported hospitalization patterns and associated costs.

This study presents, however, some limitations. As a retrospective ecological study, the data primarily reflect historical practices and outcomes, which may not fully capture future evolution of clinical practices. Additionally, while the use of the SDO database ensures a broad coverage of hospital admissions, it may lack detailed clinical information regarding the severity of CICD and its comorbidities, potentially leading to an underestimation of the complexity associated with these patients. Finally, the reliance on administrative data may introduce coding inaccuracies, which could affect the interpretation of the results.

## 5. Conclusions

CICD is still a major presence among patients in medical wards. It disproportionately affects elderly patients, leading to an increase in hospital length of stay, complications during admission, and increased direct and indirect costs. Although fewer CICD cases are treated in cardiology compared to other ward types, the costs there are significantly higher, particularly for patients undergoing advanced interventional procedures; this suggests that patients who need a cardiology admission necessitate a higher amount of resources. This may be due to their clinical presentation being more complex ab initio, or because cardiology wards are more likely to treat acute exacerbations of CICD, whereas chronic complication of CICD ends up being admitted elsewhere; this is coherent with the proportions of primary and secondary diagnoses of CICD among the investigated wards. Therefore, especially considering the aging population (in Italy, but also in the whole Western world and even in Asia), the need for a sustainable healthcare system, able to effectively manage chronic illnesses both inside and outside the hospital setting, is paramount.

Finally, our work shows evidence of the gender inequalities still present in healthcare. While CICD prevalence and risk factors are the same among men and women, the latter are less likely to receive interventional procedures than men; this suggests the presence of potential gender bias among existing treatment guidelines, as they are mostly written with the bulk of evidence coming from men-dominated clinical trials. In order to eliminate possible biases, it could be useful, in the future, to monitor the use of gender medicine both in hospital departments and in outpatient medicine. We also expect that the attention of healthcare personnel to the gender gap will be useful for a lower gender disparity, to be investigated in future publications.

## Figures and Tables

**Table 1:** Characteristics of acute admissions in internal medicine, cardiology, and geriatrics wards in Italy in 2019, by ward and by CICD diagnosis condition.

Ward Type	Diagnosis	Total Admissions	Hospitalization Days	Avg. LOS	Avg. Age
**Internal Medicine**	**CICD**	**157,417 (15.7%)**	**1,647,065**	**10.5**	**80.9**
	Principal Diagnosis	45,680 (4.6%)	449,205	9.8	81.8
	Secondary Diagnosis	111,737 (11.1%)	1,197,860	10.7	80.5
	No CICD	845,898 (84.3%)	8,356,767	9.9	73.5
**Cardiology**	**CICD**	**118,296 (25.5%)**	**806,981**	**6.8**	**71.7**
	Principal Diagnosis	56,443 (12.2%)	334,730	9.8	70.8
	Secondary Diagnosis	61,853 (13.3%)	472,251	7.6	72.5
	No CICD	345,208 (74.5%)	1,911,783	5.5	68.1
**Geriatrics**	**CICD**	**24,523 (19.4%)**	**279,525**	**11.4**	**85.3**
	Principal Diagnosis	7271 (5.7%)	78,469	10.8	85.7
	Secondary Diagnosis	17,252 (13.6%)	201,056	11.7	85.0
	No CICD	102,104 (80.6%)	1,404,724	13.8	84.2

**Table 2:** DRGs’ (Diagnosis-Related Groups’) costs of acute admissions in internal medicine, cardiology, and geriatrics wards in Italy in 2019, by ward and by CICD diagnosis condition *.

Ward Type	Diagnosis	Total Costs (EUR)	Avg. Admission Cost
**Internal Medicine**	**CICD**	**555,264,058 (15.8%)**	**3527.34**
	Principal Diagnosis	143,962,563 (4.1%)	3151.54
	Secondary Diagnosis	411,301,495 (11.7%)	3680.98
	No CICD	2,961,718,113 (84.2%)	3501.27
	TOTAL	3,516,982,171 (84.5%)	3505.36
**Cardiology**	**CICD**	**681,416,923 (27.8%)**	**5760.27**
	Principal Diagnosis	301,869,561 (12.3%)	5348.22
	Secondary Diagnosis	379,547,362 (15.5%)	6136.28
	No CICD	1,766,179,940 (72.2%)	5116.28
	TOTAL	2,447,596,863 (52.9%)	5280.64
**Geriatrics**	**CICD**	**89,282,223 (19.3%)**	**3640.75**
	Principal Diagnosis	23,340,960 (5.0%)	3210.14
	Secondary Diagnosis	65,941,263 (14.3%)	3822.24
	No CICD	372,958,862 (80.7%)	3652.73
	TOTAL	462,241,085	3650.41

* The significance of the difference between primary diagnoses with CICD and No CICD, and between secondary CICD and No CICD in each department in Table 3.

**Table 3:** Average costs difference of acute admissions in internal medicine, cardiology, and geriatrics wards in Italy in 2019, by ward and by CICD diagnosis condition.

Internal Medicine Wards		
	Primary Diagnosis (*p*-Value)	Secondary Diagnosis (*p*-Value)
**CICD vs. No CICD**	372.6 (<0.0001)	195.4 (<0.0001)
**Cardiology wards**		
	**Primary diagnosis**	**Secondary diagnosis**
**CICD vs. No CICD**	92.4 (<0.0001)	981.9 (<0.0001)
**Geriatric wards**		
	**Primary diagnosis**	**Secondary diagnosis**
**CICD vs. No CICD**	467.1 (<0.0001)	198.9 (<0.0001)

**Table 4:** Gender-based differences among admitted patients in internal medicine, cardiology, and geriatrics wards in Italy in 2019, by ward and by CICD diagnosis condition *.

Internal Medicine Wards—CICD as Primary Diagnosis							
	Total	Males			Females		
		Subtotal	65 Years	>65	Subtotal	65 Years	>65 Years
Admissions	45,609	21,490	2025	19,465	24,119	905	23,214
Admissions cost	143,744,870	68,081,073	6,547,729	61,533,344	75,663,797	2,872,865	72,790,932
Avg. admission cost	3151.7	3168.0	3233.4	3161.2	3137.1	3174.4	3135.6
Hospitalization days	448,471	205,835	16,842	188,993	242,636	8865	233,771
Avg. length of stay	9.8	9.6	8.3	9.7	10.1	9.8	10.1
Avg. n° of diagnoses	3.8	3.8	3.6	3.8	3.8	3.8	3.8
**Internal Medicine Wards—CICD as Secondary Diagnosis**							
Admissions	111,690	62,382	6727	55,655	49,308	2266	47,042
Admissions cost	411,131,752	229,293,826	24,476,707	204,817,119	181,837,926	8,420,155	173,417,771
Avg. admission cost	3681.0	3675.6	3638.6	3680.1	3687.8	3715.9	3686.4
Hospitalization days	1,197,309	651,929	65,673	586,256	545,380	24,647	520,733
Avg. length of stay	10.7	10.5	9.8	10.5	11.1	10.9	11.1
Avg. n° of diagnoses	4.6	4.6	4.4	4.7	4.6	4.6	4.6
**Cardiology Wards—CICD as Primary Diagnosis**							
	**Total**	**Males**			**Females**		
		**Subtotal**	**65 years**	**≤65 years**	**Subtotal**	**≤65 years**	**>65 years**
Admissions	56,443	39,318	13,326	25,992	17,125	3617	13,508
Admissions cost	301,869,561	221,300,664	76,700,473	144,600,191	80,568,897	18,337,969	62,230,928
Avg. admission cost	5348.2	5628.5	5755.7	5563.3	4704.8	5069.9	4607.0
Hospitalization days	334,730	218,182	63,269	154,913	116,548	19,279	97,269
Avg. length of stay	5.9	5.5	4.7	6.0	6.8	5.3	7.2
Avg. n° of diagnoses	3.0	2.9	2.6	3.0	3.1	2.6	3.3
**Cardiology Wards—CICD as Secondary Diagnosis**							
Admissions	61,853	44,501	12,399	32,102	17,352	2967	14,385
Admissions cost	379,547,362	279,212,720	79,037,497	200,175,223	100,334,642	17,535,073	82,799,569
Avg. admission cost	6136.3	6274.3	6374.5	6235.6	5782.3	5910.0	5756.0
Hospitalization days	472,251	328,313	84,678	243,635	143,938	21,882	122,056
Avg. length of stay	7.6	7.4	6.8	7.6	8.3	7.4	8.5
Avg. n° of diagnoses	4.1	4.1	3.8	4.2	4.2	3.8	4.3
**Geriatrics Wards—CICD as Primary Diagnosis**							
	**Total**	**Males**			**Females**		
		**Subtotal**	**65 years**	**>65**	**Subtotal**	**65 years**	**>65 years**
Admissions	7271	3054	38	3016	4217	17	4200
Admissions cost	23,340,960	9,930,304	140,933	9,789,371	13,410,656	71,125	13,339,531
Avg. admission cost	3210.1	3251.6	3708.8	3245.8	3180.1	4183.8	3176.1
Hospitalization days	78,469	32,749	327	32,422	45,720	174	45,546
Avg. length of stay	10.8	10.7	8.6	10.8	10.8	10.2	10.8
Avg. n° of diagnoses	3.9	3.9	4.8	3.9	3.9	4.2	3.9
**Geriatrics Wards—CICD as Secondary Diagnosis**							
Admissions	17,252	8517	113	8404	8735	42	8693
Admissions cost	65,941,263	32,606,182	477,108	32,129,074	33,335,081	148,856	33,186,225
Avg. admission cost	3822.2	3828.4	4222.2	3823.1	3816.3	3544.2	3817.6
Hospitalization days	201,056	97,570	1095	96,475	103,486	356	103,130
Avg. length of stay	11.7	11.5	9.7	11.5	11.8	8.5	11.9
Avg. n° of diagnoses	4.8	4.8	4.8	4.8	4.8	4.6	4.8

* The significance of the *p*-value between primary or secondary CICD in each ward for gender and ages in Table 5.

**Table 5:** Average length of stay and average cost of gender-based differences among admitted patients in internal medicine, cardiology, and geriatrics wards in Italy in 2019, by ward and by CICD diagnosis condition.

Internal Medicine Wards—CICD as Primary Diagnosis		
	Average Costs Difference (*p*-Value)	Average Length of Stay Difference (*p*-Value)
Males 65 years vs. Males > 65	72.2 (0.010)	1.4 (<0.0001)
Females 65 years vs. Females > 65	38.8 (0.167)	0.3 (0.249)
Males 65 years vs. Females 65 years	59.0 (0.448)	1.5 (<0.0001)
Males > 65 vs. Females > 65	25.6 (0.004)	0.4 (<0.0001)
Males vs. Females	30.9 (0.001)	0.5 (<0.0001)
**Internal Medicine wards—CICD as secondary diagnosis**		
Males 65 years vs. Males > 65	41.5 (0.110)	0.8 (<0.0001)
Females 65 years vs. Females > 65	29.4 (0.458)	0.2 (0.306)
Males 65 years vs. Females 65 years	77.3 (0.204)	1.1 (<0.0001)
Males > 65 vs. Females > 65	6.3 (0.591)	0.5 (<0.0001)
Males vs. Females	12.2 (0.298)	0.6 (<0.0001)
**Cardiology wards—CICD as primary diagnosis**		
	**Costs**	**Hospitalization days**
Males 65 years vs. Males > 65	197.6 (<0.0001)	1.2 (<0.0001)
Females 65 years vs. Females > 65	468.6 (<0.0001)	1.9 (<0.0001)
Males 65 years vs. Females 65 years	692.2 (<0.0001)	0.6 (<0.0001)
Males > 65 vs. Females > 65	963.2 (<0.0001)	1.2 (<0.0001)
Males vs. Females	931.2 (<0.0001)	1.3 (<0.0001)
**Cardiology wards—CICD as secondary diagnosis**		
Males 65 years vs. Males > 65	138.9 (0.025)	0.8 (<0.0001)
Females 65 years vs. Females > 65	154.1 (0.206)	1.1 (<0.0001)
Males 65 years vs. Females 65 years	464.5 (<0.0001)	0.5 (<0.0002)
Males > 65 vs. Females > 65	479.6 (<0.0001)	0.9 (<0.0001)
Males vs. Females	492.0 (<0.0001)	0.9 (<0.0001)
**Geriatrics wards—CICD as primary diagnosis**		
	**Costs**	**Hospitalization days**
Males 65 years vs. Males > 65	463.0 (0.243)	2.1 (0.010)
Females 65 years vs. Females > 65	1007.7 (0.215)	0.6 (0.735)
Males 65 years vs. Females 65 years	475.1 (0.546)	1.6 (0.314)
Males > 65 vs. Females > 65	69.7 (0.001)	0.1 (0.600)
Males vs. Females	71.4 (<0.0001)	0.1 (0.508)
**Geriatrics wards—CICD as secondary diagnosis**		
Males 65 years vs. Males > 65	399.1 (0.399)	1.8 (0.034)
Females 65 years vs. Females > 65	273.4 (0.581)	3.4 (0.010)
Males 65 years vs. Females 65 years	678.0 (0.415)	1.2 (0.427)
Males > 65 vs. Females > 65	5.5 (0.861)	0.4 (0.004)
Males vs. Females	12.1 (0.702)	0.4 (0.003)

## Data Availability

The datasets presented in this article are not readily available because they are extracted by the national SDO Data that are available only after special permission. Requests to access the datasets should be directed to CREA Sanità, Federico Spandonaro.
